# Measurement properties of the EQ-5D in children and adolescents: a systematic review protocol

**DOI:** 10.1186/s13643-023-02443-7

**Published:** 2024-01-05

**Authors:** Caique de Melo do Espirito Santo, Verônica Souza Santos, Gisela Cristiane Miyamoto, Alessandro Chiarotto, Marisa Santos, Tiê Parma Yamato

**Affiliations:** 1 Master’s and Doctoral Programs in Physical Therapy, Universidade Cidade de São Paulo Rua Cesário Galeno, 448/475, São Paulo, Tatuapé 03071-000 Brazil; 2 Department of Health Sciences, Faculty of Science, Vrije Universiteit Amsterdam, Amsterdam Public Health, Amsterdam, The Netherlands; 3 Department of General Practice, Erasmus MC, University Medical Center, Rotterdam, The Netherlands; 4 Instituto Nacional de Cardiologia, Rio de Janeiro, Brazil; 5 School of Public Health, Faculty of Medicine and Health, Institute for Musculoskeletal Health, The University of Sydney, Sydney, Australia; 6 Center for Pain, Health and Lifestyle, Sydney, Australia

**Keywords:** Measurement properties, Children and adolescents, EQ-5D, Health-related quality of life

## Abstract

**Background:**

Although the EQ-5D instruments have been initially designed for adult populations, there are new studies evaluating and applying these instruments to children and adolescents. The EuroQol Group adapted and created two versions designed for these groups, i.e., the EQ-5D-Y versions. The measurement properties of the EQ-5D have been systematically reviewed in different health conditions. However, there is a lack of a proper systematic assessment including the studies’ risk of bias and focusing on recent studies assessing the EQ-5D instruments in children and adolescents. The lack of a systematic assessment of the EQ-5D versions does not allow us to have a comprehensive evaluation of the validity, reliability, and responsiveness of these instruments among children and adolescents. This systematic review aims to critically appraise and summarize the evidence on the measurement properties of the EQ-5D instruments (self-reported version – answered by children and adolescents; and proxy versions – versions reported by parents, caregivers, or health professionals) in children and adolescents.

**Methods:**

A systematic review searching the following electronic databases: MEDLINE, EMBASE, CINAHL, EconLit, National Health Service Economic Evaluation Database (NHS-EED), Health Technology Assessment (HTA) database. Two independent reviewers will screen titles and abstracts and select full texts for eligibility. The COnsensus-based Standards for the selection of health Measurement INstruments (COSMIN) methodology will be followed to conduct three main assessment steps: risk of bias, quality criteria for measurement properties, and evidence synthesis.

**Discussion:**

This systematic review will provide comprehensive information about the evidence regarding the measurement properties of EQ-5D instruments in children and adolescents of different settings and countries.

**Systematic review registration:**

Open Science Framework with Registration https://osf.io/r8kt9/ and PROSPERO: CRD42020218382.

**Supplementary Information:**

The online version contains supplementary material available at 10.1186/s13643-023-02443-7.

## Background

The development of health-related quality of life (HRQoL) measurements in children and adolescents have increased in the last twenty years [1]. In general, HRQoL measurements are multidimensional and include physical, psychological, and social functioning domains [1, 2]. Most of the instruments aiming to assess this domain are self-reported and, consequently, refer to self-perceived HRQoL [2]. HRQoL instruments usually target two different methods of measurement: 1) an indirect measurement assessing the quality of life as a health status (i.e., measured by specific or generic questionnaires); or 2) a value of health status to generate both health profiles and index values (i.e., using the utility score measured by the EQ-5D for example) [2].

The EQ-5D instruments are well-known, widely used in the literature, and have been translated into more than 170 languages [3]. The EQ-5D responses can be interpreted in three different manners: 1) descriptively by using the health profiles of the individual items (e.g., 11,111 for the descriptive system); 2) country-specific index values estimated from preference weights of the different health states (this index usually ranges from 0 – representing “dead” – to 1 – representing “full health”; 1 in this case also represents the best level of severity or no severity in the five dimensions [11111] of the EQ-5D). This country-specific index is known as the value set of the EQ-5D for each country and it should be developed from a valuation study (e.g., composite time trade-off and discrete choice experiment) according to the recommendations from the EuroQol Group. The data should be collected from the general population in each country/region; and 3) self-rated health status measures (e.g., using the EQ-5D visual analogue scale [EQ-VAS]) [4]. A recent review of national health technology assessment guidelines showed that the EQ-5D was the most commonly used instrument in cost-utility analysis within economic evaluations [5].

The original 3-level version of EQ-5D (EQ-5D-3L) was developed in the 90's and, after its growth in use, the EuroQol Group developed in 2009 the five-level version (EQ-5D-5L) in order to improve its sensitivity and reduce ceiling effects [3, 6, 7]. After decades from the EQ-5D initial publication, the EuroQol Group adapted and created two versions specifically designed for children and adolescents, known as EQ-5D-Y-3L and EQ-5D-Y-5L [3, 8, 9]. For both instruments, there is the self-reported version (answered by children and adolescents) and a proxy version (generally reported by parents, caregivers or health professionals) [10]. The measurement properties of these instruments have been tested in children and adolescents with different health conditions (such as, type 1 diabetes mellitus, asthma, general population, psychiatric disorders, and idiopathic scoliosis) [11–16]. One systematic literature review evaluated the measurement properties of the EQ-5D instruments (EQ-5D adult version, EQ-5D-Y-3L, Dutch EQ-5D child version and, an extended questionnaire with cognitive dimension EQ-5D + C) in children and adolescents, but without assessing the risk of bias of the included studies [17]. This review included studies between 1999 and 2010, and a large amount of new studies of the EQ-5D-Y-3L [12, 17–24] have been published in the last ten years, including studies focusing on the more recent version, the EQ-5D-Y-5L [8, 14–16, 25]. Moreover, the COnsensus-based Standards for the selection of health Measurement INstruments (COSMIN) initiative developed a recent methodological guideline for systematic reviews of patient-reported outcome measures (PROMs) like the EQ-5D, including a tool to assess the risk of bias of studies on measurement properties and to evaluate the quality of development of patient-reported outcome measures [26–28]. The lack of a systematic review with recent data assessing the risk of bias of the studies and the lack of a systematic assessment of the newly EQ-5D-Y versions does not allow to have a comprehensive picture on the validity, reliability and responsiveness of these instruments among children and adolescents.

## Objective

The aim of this systematic review is to critically appraise and summarize the evidence on the measurement properties of the EQ-5D instruments (self-reported version – answered by children and adolescents; and, proxy versions – versions reported by parents, caregivers, or health professionals) in children and adolescents.

## Methods

This protocol was registered on the Open Science Framework (OSF) https://osf.io/r8kt9/. This protocol also was registered on International Prospective Register of Systematic Reviews (PROSPERO) with registration number CRD42020218382. This review has been prepared following the Preferred Reporting Items for Systematic Reviews and Meta-Analysis Protocol (PRISMA-P) for the reporting, and the COSMIN guidance in the conduction [27, 29]. The details of PRISMA-P Checklist are presented in an Additional file 1.

### Measurement instruments of interest

The instruments of interest are the EQ-5D instruments developed by the EuroQol Group, including adult and children versions: EQ-5D-3L, EQ-5D-5L, EQ-5D-Y-3L, and EQ-5D-Y-5L [6–9]. We decided to include the adult versions of the EQ-5D (EQ-5D-3L and EQ-5D-5L) because both were the first instruments of the EuroQol Group to be created and already had their measurement properties tested in children and adolescents [3, 18, 19, 30–32]. The evaluation of the EQ-5D instruments in all languages and with various modes of administration is of interest [3]

The EQ-5D-3L was developed for the adult population and it measures HRQoL in five dimensions: mobility, self-care, usual activities, pain/discomfort, and anxiety/depression. Each dimension has three levels of severity: ‘no problems’, ‘some problems’, and ‘extreme problems’ [4]. The EQ-5D-5L was also developed for the adult population and also measures HRQoL in the same five dimensions, however, each dimension has five levels of severity: ‘no problems’, ‘slight problems’, ‘moderate problems’, ‘severe problems’, and ‘extreme problems’ [6]. Both instruments can provide a health state of a descriptive system (e.g., 11,111 – meaning no problems in all dimensions), which can be converted to an index value (ranging from 0 – representing “dead” – to 1 – representing “full health”) of the general population of a country. Both instruments also include a visual analogue scale (EQ-VAS) ranging from 0 to 100, in which, zero represents the worse health-perceived status and 100 represents the best health-perceived status.

The EQ-5D-Y-3L and EQ-5D-Y-5L were created based on the EQ-5D-3L and EQ-5D-5L, respectively [8, 9]. Both instruments have a self-reported version (answered by children and adolescents) and proxy versions (reported by parents, caregivers, or health professionals when children are unable to report their HRQoL) and have been developed specifically for children and adolescents aged 8–15 years. Both self-reported versions (EQ-5D-Y-3L and EQ-5D-Y-5L) had language modifications to warrant better understanding by children and adolescents, but both have the same structure as the original versions of the instrument.

### Eligibility criteria

We will include studies that measured HRQoL through one or more EQ-5D instruments in children and adolescents up to 19 years old (or at least 80% of the sample with mean age up to 19 years old). We will include studies that used either the self-reported version (answered by children and adolescents) or proxy versions (reported by parents, caregivers, or health professionals when children are unable to report their HRQoL) of the instruments, and that tested at least one measurement property in the study (e.g., internal consistency, reliability, responsiveness). We will include any types of studies of measurement properties/clinimetric (e.g., cross-sectional and cohort with test–retest). There will be no restrictions on the type of setting or health conditions of the population.

We will include studies from 1990 because it was in this period that the EuroQol Group published the first version of the EQ-5D [7]. There will be no restrictions on language, and we will only include full-text articles published in peer-review journals (we will not include any grey literature).

### Literature search

We will perform the search strategy on the following databases: MEDLINE, EMBASE, CINAHL, EconLit, National Health Service Economic Evaluation Database (NHS-EED), and Health Technology Assessment (HTA) databases. To ensure a comprehensive search, we will conduct manual searches on EuroQol Research Foundation Website for potentially eligible studies [33]. We will also screen the reference lists of the included studies for other potentially eligible studies. In addition, we will search for studies meeting our inclusion criteria among the studies included in a previous systematic review [17].

### Search strategy

The search strategy of this systematic review will be created based on three key elements, according to the COSMIN recommendations: 1) ‘children and adolescents’; 2) ‘EQ-5D’; and 3) a sensitive filter to identify studies about measurement properties of EQ-5D instruments [27]. The filter to identify studies of measurement properties is highly sensitive and it was previously validated [34]. The details of the search strategy on MEDLINE are presented in an Additional file 2.

### Study selection

The results from the searches will be uploaded and managed in the EndNote Software version X9 for the removal of duplicates [35]. Two review authors (CMES and VSS) will independently screen all titles and abstracts, and also the full texts for potentially eligible studies. Any disagreement will be discussed and, if consensus cannot be reached, a third review author (TPY or GCM) will make a decision. Screening and data extraction will be performed using DistillerSR (Evidence Partners, Ottawa, Canada).

### Data extraction, data items and outcomes

Two authors (CMES and VSS) will independently extract the data for each eligible study. Disagreements between the reviewers will be discussed and a third reviewer (TPY or GCM) will be contacted when a consensus cannot be reached. If necessary, we will contact the study’s authors to ask for additional information not reported in the article. We will make three attempts through email, over a period of one month. Two authors (TPY and GCM) with experience in conducting systematic reviews developed a data extraction form, and this form was based on those used to conduct COSMIN systematic reviews [27]. The main outcome of interest is any measurement properties of the EQ-5D instruments (self-reported or proxy versions) used to measure HRQoL in children and adolescents. We will extract the following data:

- EQ-5D version (e.g., EQ-5D-3L);
- Scores used (e.g., VAS, utility score);
- Reference, year;
- Language, country;
- Population (sample size, female [%], age, description of health condition);
- Context of use;
- Mode of administration (e.g., self-report, parent/proxy report);
- Number of missing data and response rates;
- Measurement properties of interest (e.g., internal consistency, reliability, responsiveness) according to the COSMIN taxonomy;
- Results of measurement properties;
- Feasibility and interpretability data;
- Recall period.

### Evaluation of measurement properties and specific assessment of content validity

Measurement properties will be defined in line with the COSMIN taxonomy for PROMs [36]. Their evaluation will be performed through three components: 1) the risk of bias of the individual studies (methodological quality); 2) the application of quality criteria for (in)sufficient measurement properties; and 3) the Grading of Recommendations Assessment, Development, and Evaluation (GRADE) assessment to rate the quality of evidence for each measurement property of each instrument (evidence synthesis) [26–28]. All of these three components will be assessed by two authors (CMES and VSS). The disagreements between the reviewers will be discussed and a third reviewer (TPY or GCM) will be contacted when a consensus cannot be reached.

#### Risk of bias

The risk of bias assessment of the included articles will be performed through the COSMIN Risk of Bias checklist for PROMs [28]. The checklist has standards referring to design requirements and preferred statistical methods to evaluate the methodological quality of studies on measurement properties [27]. According to this checklist, the first step of the assessment is to identify each measurement property assessed in the study (i.e., one study can describe one or more different measurement properties) according to the COSMIN taxonomy of measurement properties [36].

For each measurement property, there is a COSMIN box with all standards needed to assess the quality of a study on that specific measurement property. The COSMIN Risk of Bias Checklist encompasses ten boxes with standards for PROM development (box 1) and for nine measurement properties: content validity (box 2); structural validity (box 3); internal consistency (box 4); cross‐cultural validity\measurement invariance (box 5); reliability (box 6); measurement error (box 7); criterion validity (box 8); hypotheses testing for construct validity (box 9) and responsiveness (box 10) [28].

Content validity is assessed through the use of boxes 1 and 2, as the original studies for developing the instruments are necessary to assess this measurement property [26]. The content validity will be scored separately (steps 1 and 2 of the COSMIN methodology for assessing the content validity of PROMs) in order to: 1) assess the quality of the PROM development, using COSMIN box 1 (considering the concept elicitation and the cognitive interview); and 2) assess the quality of content validity studies on the PROM (if available), using COSMIN box 2 (according to the relevance, comprehensiveness, and comprehensibility from professionals and patients) [26].

The internal structure is evaluated by the content in boxes 3 to 5, and boxes 6 to 10 address the remaining measurement properties. As in one article, one or more different measurement properties can be studied, the COSMIN Risk of Bias checklist is a modular tool. This means that each measurement property studied should be assessed separately according to each corresponding box. It will not be necessary to complete the whole checklist when evaluating the methodological quality of an included study, instead, the measurement property evaluated in each article will determine which boxes need to be completed. For example, if an article evaluates internal consistency, construct validity, and reliability, then three boxes needed to be completed (i.e., box 4 to internal consistency, box 9 to construct validity, and box 6 to reliability).

Each standard within a COSMIN box will be rated in a four-point rating system as ‘very good’, ‘adequate’, ‘doubtful’, or ‘inadequate’ methodological quality [28]. To determine an overall score for each study the lowest rating of any standard in the correspondent boxes will need to be taken into consideration [37]. For example, if there is one standard rated as ‘adequate’ and another rated as ‘inadequate’, then the measurement property represented by this box will have an overall score of ‘inadequate’ methodological quality. This overall score of the risk of bias will be then used to grade the quality of the evidence (for each measurement property) [38]. In line with the COSMIN user manual, since the development studies of the EQ-5D-3L and the EQ-5D-5L were already scored in a previous systematic review, those ratings will be adopted in this review [39, 40].

Conventional psychometric tests of validity in the generic preference-based measures for use in economic evaluation can be considered inappropriate [41]. Thus, we will complement our methodological quality assessment using a checklist to describe the practically, reliability and validity for judging the appropriateness of the EQ-5D of the included studies, which incorporates economists’ notion of preferences. This checklist is composed of: practicality (three items); reliability (four items); validity subdivided into three parts: description (three items); valuation (four items); and empirical (one item) [41]. If one study tested reliability, then we will complete the four items of reliability on this checklist. This checklist can be found elsewhere [41].

#### Quality criteria for measurement properties

We will also assess the quality of all measurement properties of each included study using quality criteria [42, 43]. For each measurement property studied (in each included study), a criterion is defined as: sufficient ( +), insufficient (-), or indeterminate (?) [27].

#### Evidence synthesis

The quality of evidence will be measured through a modified version of the GRADE [27]. In line with the GRADE approach for systematic reviews of intervention studies, the quality of the evidence will be graded as high, moderate, low, or very low evidence. The results of each EQ-5D instrument (EQ-5D-3L, EQ-5D-5L, EQ-5D-Y-3L, and EQ-5D-Y-5L) will be assessed individually [44].

A rating system was developed specifically to summarize the quality of the evidence for content validity, step 3 of the COSMIN methodology for assessing the content validity of PROMs [26]. It consists on rating the studies' results of the PROM development and content validity following ten criteria. Then we will summarize all available evidence and grade the quality of the evidence. This rating system was divided in three steps:

- Rating the result of the single studies on PROM development and content validity (if available) against the 10 criteria for good content validity (5 on relevance, 1 on comprehensiveness, 4 on comprehensibility). The ratings will range as sufficient ( +), insufficient (-) or indeterminate (?);
- The results of the available studies are qualitatively summarized to determine whether overall, the relevance, comprehensiveness, comprehensibility, and overall content validity of the PROM is sufficient ( +), insufficient (–), or inconsistent ( ±). We will determine if the overall content validity of the PROM is sufficient or insufficient. The overall rating will be sufficient or insufficient if all the ratings per study were sufficient or insufficient, respectively. If the ratings present inconsistency between studies, we will explain the reasons for inconsistency and summarize the overall rating of the studies per subgroup. If no explanation is found, the overall rating will be determined as inconsistent ( ±);
- The overall ratings (relevance, comprehensiveness, comprehensibility, and content validity) will be accompanied by grading for the quality of the evidence. The evidence can be high, moderate, low, or very low. We will use the GRADE depending on the presence of three domains: risk of bias, inconsistency, and indirectness. We will start assuming that there is high-quality evidence for content validity. The levels of quality of evidence will be downgraded with one or more levels (to moderate, low, or very low) if there is a (serious or very serious) risk of bias, unexplained inconsistency in results, and/or indirect findings. We will follow a minimized version of the flow chart as additional guidance that can be consulted elsewhere [26].

Regarding the other measurement properties, the results of all available studies on a measurement property can be quantitatively pooled or qualitatively summarized. We will use the criteria for good measurement properties to determine whether overall the measurement properties of the EQ-5D instruments are sufficient ( +), insufficient (-), inconsistent ( ±) or indeterminate (?) [27]. We will then determine if the included studies are consistent or inconsistent [38]. Studies will be considered consistent if they showed at least 75% of the results of good measurement properties in accordance with each measurement property, i.e., measurement error insufficient (-) for at least 75% of cases. If the studies are consistent (at least 75% of the results sufficient or insufficient), the results of each measurement property will be pooled in a random effect meta-analysis. The pooled estimates will be assessed by calculating weighted means and 95% confidence intervals [27]. If the studies are inconsistent, we will try to explain the reasons for inconsistency and summarize per subgroup. In this case, we will qualitatively summarize the results. The results will be shown by range (lowest and highest) of values or percentage of confirmed hypotheses (when construct validity) [27]. If no explanation is found, the overall rating will be classified as inconsistent ( ±). If not enough information is available, the overall rating will be classified as indeterminate (?) [27].

Previously, we formulated a hypothesis to construct validity and responsiveness [27]. Related construct validity: 1) if the instruments measure a similar construct the correlation should be ≥ 0.50; 2) if measure a related, but not similar construct the correlation should be ≥ 0.30 and < 0.50; and 3) if instruments measure an unrelated construct the correlation should be < 0.30 (instruments that do not measure HRQoL in children and adolescents). Related to the responsiveness, we hypothesized an area under the curve (AUC) ≥ 0.70 (e.g., global perceived effect scales) [27].

For these other measurement properties, the downgrading in GRADE will be applied depending on the presence of four factors (instead of five): 1) risk of bias of the studies will be downgraded one level if there is serious risk of bias (i.e., multiple studies of doubtful quality available, or if there is only one study with adequate quality), two levels if there is very serious risk of bias (multiple studies of inadequate quality, or there is only one study with doubtful quality available, or three levels if there is extremely risk of bias – only one study with inadequate quality available); 2) inconsistency, i.e., unexplained inconsistency of results across studies (downgraded if the most studies was inconsistent according good measurement properties, we will decided if is necessary downgraded one or two levels according inconsistent found); 3) imprecision, i.e., total sample size of the available studies (downgraded one level if the total sample is below 100 and two level if the total sample is below 50); and 4) indirectness, i.e., evidence from different populations than the population of interest in the review (downgraded if the sample of studies will be very different, we will decided if is necessary downgraded one or two levels according heterogeneity found) [27].

### Data synthesis

We will provide an overall quality criteria for good measurement properties (i.e., sufficient ( +), insufficient (-), inconsistent ( ±) or indeterminate (?)) as well as the respectively certainty of evidence (i.e., high, moderate, low and very low). The results of evidence synthesis will be categorized for each version of the EQ-5D instruments (EQ-5D-3L, EQ-5D-5L, EQ-5D-Y-3L, EQ-5D-Y-5L) in order to provide the best version of the instrument for children and adolescents. In addition, we will categorize each version of the instruments according to the mode of administration (i.e., self-report or proxy-report).

## Discussion

### Purpose

The aim of this systematic review is to critically appraise and summarize the evidence on measurement properties of the EQ-5D instruments (self- and proxy-reported version) in children and adolescents. Although similar systematic reviews have been published [17, 45, 46], there is no clear information on the literature regarding the risk of bias of the studies on measurement properties of the EQ-5D instruments used for children and adolescents. In addition, there is no detailed and robust assessment of the content validity for newly instruments (especially the EQ-5D-Y-3L and the EQ-5D-Y-5L), no information regarding other measurement properties (i.e., reliability, validity and responsiveness), and no information on the quality criteria and certainty of evidence following the COSMIN methodology [27].

### Strengths and limitations

This systematic review has strengths and limitations. A strength of this systematic review is that we will assess the risk of bias in the included studies using the COSMIN Risk of Bias Checklist [28]. One systematic literature review of the EQ-5D (EQ-5D adult version, EQ-5D-Y-3L, Dutch EQ-5D child version, and, an extended questionnaire with cognitive dimension EQ-5D + C) in children and adolescents conducted in 2011 did not assess the risk of bias [17]. Therefore, it is necessary to understand the methodological quality of these studies. Another strength of this systematic review is to perform an update of this scenario of the EQ-5D instruments, and the use of the EQ-5D instruments among children and adolescents due to several studies that were published over 10 years. Studies have tested measurement properties of adult versions EQ-5D-3L and EQ-5D-5L in children and adolescents, and specific versions such as EQ-5D-Y-3L and EQ-5D-Y-5L [11, 12, 14, 16, 18, 19, 23, 25, 47, 48]. The results of this study will summarize the evidence of the measurement properties of the EQ-5D and their different interpretations (descriptive system, EQ-VAS, utility score) among children and adolescents. This systematic review is strengthened also by the adherence to the recommendations from PRISMA-P for the reporting, and the methodology COSMIN to conduct a systematic review of measurement properties of the EQ-5D instruments [27, 29, 36]. Our aim is specifically to assess the measurement properties of the EuroQol instruments among children and adolescents, but we believe a limitation can be the inclusion of only EQ-5D instruments. There are other generic instruments that aim to measure HRQoL in the youth population, such as the Pediatric Quality of Life Inventory (PedsQL), KIDSCREEN for example, as well as, the generic preference-based measures for children, such as the Child Health Utility 9D (CHU9D) for example [49–51]. Another limitation of this study is that although the EQ-5D instruments have been considered as a PROM for HRQoL, the EQ-5D instruments have their features in line with generic preference-based measures [52, 53]. Despite the literature showing that COSMIN guidelines can be limited to assess generic preference-based measures, we choose to base our assessment of measurement properties of the EQ-5D instruments using this guideline because there is no gold standard to assess generic preference-based measures [27]. Thus, in order to complement our assessment, we decided to add the use of a few measurement properties covered by COSMIN, but according to a specific checklist to describe the generic preference-based measures of HRQoL instruments [41].

### Implications for practice and research

The clinical relevance of the EQ-5D (especially EQ-5D-Y-3L and EQ-5D-Y-5L) is to assess HRQoL and health-perceived status in children and adolescents aged 8–15 years. Furthermore, the EQ-5D can facilitate the calculation of the quality-adjusted life of years (QALYs) through the country-specific index values for example, that are used in economic evaluations. The EQ-5D instruments are useful to assess the HRQoL outcome in different design studies (e.g., cross-sectional, cohort, randomized controlled trial), in economic evaluation studies (e.g., cost-effectiveness, cost-utility analysis), and also in clinical practice by health professionals [3]. Thus, this systematic review will provide updated evidence on the measurement properties of EQ-5D in children and adolescents with several health conditions conducted in different cultural contexts and countries. This will help the choice between EQ-5D versions based on their measurement properties results and overall quality of evidence. It will also provide the risk of bias in studies and overall quality evidence of EQ-5D instruments in children and adolescents that, to our knowledge, is not available at the moment. In addition, an updated systematic review will help to disseminate the importance of the EQ-5D instruments in children and adolescents and to signalize the gap to guide future measurement properties studies.

### Supplementary Information

**Additional file 1.**

** Additional file 2.**

## Data Availability

Not applicable.

## Abbreviations

HRQoL: Health-related quality of life
EQ-VAS: EQ-5D visual analogue scale
COSMIN: COnsensus-based Standards for the selection of health Measurement Instruments
OSF: Open Science Framework
PROSPERO: International Prospective Register of Systematic Reviews
PRISMA-P: Preferred Reporting Items for Systematic Reviews and Meta-Analysis Protocol
MEDLINE: Medical Literature Analysis and Retrieval System Online
EMBASE: Excerpta Medica database
CINAHL: Cumulative Index of Nursing and Allied Health Literature
NHS-EED: National Health Service Economic Evaluation Database
HTA: Health Technology Assessment
GRADE: Grading of Recommendations Assessment, Development, and Evaluation
PROMs: Patient-reported outcome measures
AUC: Area under the curve
PedsQL: Pediatric Quality of Life Inventory
CHU9D: Child Health Utility 9D
QALYs: Quality-adjusted life of years
