# Supplementary material for: Measurement properties of the EQ-5D in children and adolescents: a systematic review protocol
Source: Syst Rev. 2024 Jan 5;13:18. doi: 10.1186/s13643-023-02443-7 (PMC10768350; doi:10.1186/s13643-023-02443-7)
Supplement: Supplementary file 2 — Additional file 2. [file 13643_2023_2443_MOESM2_ESM.pdf]

Additional file 2

Search strategy on MEDLINE:

Population: children and adolescents

1 Exp child/

2 Child*.tw.

3 Exp adolescent/

4 Adolescen*.tw.

5 Youth.tw.

6 Exp child, preschool/

7 School.tw.

8 Student.tw.

9 Teena*.tw.

10 Young.tw.

11 pediatric.tw.

12 1 OR 2 OR 3 OR 4 OR 5 OR 6 OR 7 OR 9 OR 10 OR 11

Type of instrument(s): EQ-5D

13 EuroQol.tw.

14 EQ-5D.tw.

15 EQ-5D-3L.tw.

16 EuroQol 5-dimension.tw.

17 EuroQol 5-Dimension 5-Level.tw.

18 EuroQol-Five*.tw.

19 EQ-5D-5L.tw.

20 EuroQol-Five dimension.tw.

21 EQ-5D-3L.tw.

22 EQ-5D-3L-Y.tw.

23 EuroQol-5D-Youth.tw.

24 EQ-5D Youth.tw.

25 EQ-5D-Y-3L.tw.

26 EQ-5D-Y-5L.tw.

27 EQ-5D-5L-Y.tw.

28 EQ-5D*.tw.

29 13 OR 14 OR 15 OR 16 OR 17 OR 18 OR 19 OR 20 OR 21 OR 22 OR 23 OR 24 OR 25 OR 26 OR 27 OR 28

Measurement properties filter:

(instrumentation.fs. OR methods.fs. OR Validation Studies.pt. OR Comparative Study.pt. OR exp psychometrics/ OR psychometr*.ti,ab. OR clinimetr*.mp. OR clinometr*.mp. OR exp outcome assessment (health care)/ OR outcome assessment.ti,ab. OR outcome measure*.mp. OR exp observer variation/ OR observer variation.ti,ab. OR exp Health Status Indicators/ OR exp reproducibility of results/ OR reproducib*.ti,ab. OR exp discriminant analysis/ OR reliab*.ti,ab. OR unreliab*.ti,ab. OR valid*.ti,ab. OR coefficient of variation.ti,ab. OR coefficient.ti,ab. OR homogeneity.ti,ab. OR homogeneous.ti,ab. OR internal consistency.ti,ab. OR (cronbach*.ti,ab. AND (alpha.ti,ab. OR alphas.ti,ab.)) OR (item.ti,ab. AND (correlation*.ti,ab. OR selection*.ti,ab. OR reduction*.ti,ab.)) OR agreement.mp. OR precision.mp. OR imprecision.mp. OR precise values.mp. OR test-retest.ti,ab. OR (test.ti,ab. AND retest.ti,ab.) OR (reliab*.ti,ab. AND (test.ti,ab. OR retest.ti,ab.)) OR stability.ti,ab. OR interrater.ti,ab. OR inter-rater.ti,ab. OR intrarater.ti,ab. OR intra-rater.ti,ab. OR intertester.ti,ab. OR inter-tester.ti,ab. OR intratester.ti,ab. OR intra-tester.ti,ab. OR interobserver.ti,ab. OR inter-observer.ti,ab. OR intraobserver.ti,ab. OR intra-observer.ti,ab. OR intertechnician.ti,ab. OR inter-technician.ti,ab. OR intratechnician.ti,ab. OR intra-technician.ti,ab. OR interexaminer.ti,ab. OR inter-examiner.ti,ab. OR intraexaminer.ti,ab. OR intra-examiner.ti,ab. OR interassay.ti,ab. OR inter-assay.ti,ab. OR intraassay.ti,ab. OR intra-assay.ti,ab. OR interindividual.ti,ab. OR

inter-individual.ti,ab. OR intraindividual.ti,ab. OR intra-individual.ti,ab. OR interparticipant.ti,ab. OR inter-participant.ti,ab. OR intraparticipant.ti,ab. OR intra-participant.ti,ab. OR kappa.ti,ab. OR kappa's.ti,ab. OR kappas.ti,ab. OR repeatab*.mp. OR ((replicab*.mp. OR repeated.mp.) AND (measure.mp. OR measures.mp. OR findings.mp. OR result.mp. OR results.mp. OR test.mp. OR tests.mp.)) OR generaliza*.ti,ab. OR generalisa*.ti,ab. OR concordance.ti,ab. OR (intraclass.ti,ab. AND correlation*.ti,ab.) OR discriminative.ti,ab. OR known group.ti,ab. OR factor analysis.ti,ab. OR factor analyses.ti,ab. OR factor structure.ti,ab. OR factor structures.ti,ab. OR dimension*.ti,ab. OR subscale*.ti,ab. OR (multitrait.ti,ab. AND scaling.ti,ab. AND (analysis.ti,ab. OR analyses.ti,ab.)) OR item discriminant.ti,ab. OR interscale correlation*.ti,ab. OR error.ti,ab. OR errors.ti,ab. OR individual variability.ti,ab. OR interval variability.ti,ab. OR rate variability.ti,ab. OR (variability.ti,ab. AND (analysis.ti,ab. OR values.ti,ab.)) OR (uncertainty.ti,ab. AND (measurement.ti,ab. OR measuring.ti,ab.)) OR standard error of measurement.ti,ab. OR sensitiv*.ti,ab. OR responsive*.ti,ab. OR (limit.ti,ab. AND detection.ti,ab.) OR minimal detectable concentration.ti,ab. OR interpretab*.ti,ab. OR ((minimal.ti,ab. OR minimally.ti,ab. OR clinical.ti,ab. OR clinically.ti,ab.) AND (important.ti,ab. OR significant.ti,ab. OR detectable.ti,ab.) AND (change.ti,ab. OR difference.ti,ab.)) OR (small*.ti,ab. AND (real.ti,ab. OR detectable.ti,ab.) AND (change.ti,ab. OR difference.ti,ab.)) OR meaningful change.ti,ab. OR ceiling effect.ti,ab. OR floor effect.ti,ab. OR Item response model.ti,ab. OR IRT.ti,ab. OR Rasch.ti,ab. OR Differential item functioning.ti,ab. OR DIF.ti,ab. OR computer adaptive testing.ti,ab. OR item bank.ti,ab. OR cross-cultural equivalence.ti,ab.)

12 AND 29 AND measurement properties filter
